# Amyloid-beta statuses prediction with free water MR imaging features in Alzheimer’s disease using machine learning models

**DOI:** 10.1186/s12880-026-02380-6

**Published:** 2026-04-27

**Authors:** Rongshen Zhou, Xuan Sun, Siyu Chen, Shilong Zhao, Cui Zhao, Junda Qu, Weiyi Zeng, Chunlin Li, Xu Zhang, Zihan Li, Yiqing Wang, Ting Zhang, Xian Xu, Jianjun Jia, Ying Liang

**Affiliations:** 1 School of Biomedical Engineering, Capital Medical University, Beijing, China; 2 Department of Neurology, The Second Medical Centre, National Clinical Research Center of Geriatric Diseases, Chinese PLA General Hospital, Beijing, China; 3 Medical School of Chinese PLA, Beijing, China; 4 Cognitive Science and Allied Health School, Beijing Language and Culture University, Beijing, China; 5 School of Artificial Intelligence, Beijing University of Posts and Telecommunications, Beijing, China; 6 Department of Radiology & Precision and Intelligence Medical Imaging Lab, Beijing Friendship Hospital, Capital Medical University, Beijing, China; 7 Population Health Sciences, German Centre for Neurodegenerative Diseases, Bonn, Germany; 8 Institute of Geriatrics, The Second Medical Centre, National Clinical Research Center of Geriatric Diseases, Chinese PLA General Hospital, Beijing, China; 9 Department of Radiology, The Second Medical Centre, National Clinical Research Center of Geriatric Diseases, Chinese PLA General Hospital, Beijing, China

**Keywords:** Alzheimer’s disease, Machine learning, Free water diffusion tensor imaging, Amyloid-beta, Classification model

## Abstract

**Purpose:**

This study aimed to identify the effectiveness of free water MRI (FW-MRI) features for predicting amyloid-beta (Aβ) statuses in Alzheimer’s disease (AD) by constructing diagnostic models using machine learning analysis.

**Methods:**

This study retrospectively included 96 patients of mild cognitive impairment (MCI) and AD (69 Aβ-positive and 27 Aβ-negative). Clinical characteristics, FW-corrected and standard diffusion indices, and structural MRI indices were collected. Three supervised machine learning algorithms, including random forest (RF), support vector machine (SVM), and extreme gradient boosting (XGB), were adopted to construct a diagnostic model for distinguishing Aβ deposition in AD. SHapley Additive exPlanation (SHAP) value was used as an interpretable algorithm to identify influential characteristics based on the best-performing model.

**Results:**

In the single-modality model, FW-DTI achieved better classification performance than conventional DTI, which obtained accuracies all above 80% among three machine learning approaches on the internal dataset (RF = 0.800, SVM = 0.867, XGB = 0.800). In the multi-modality model, the XGB model integrated FW-DTI, voxel-based morphometry, and clinical features outperformed the RF and SVM models, achieving an accuracy of 86.7% and an area under the curves (AUC) value 93.2% in the training cohort, and an accuracy of 77.8% and AUC value of 83.1% in the external testing cohort. The model demonstrated high sensitivity but relatively low specificity, indicating a tendency toward positive predictions. Furthermore, FW-DTI indices were shown to have the highest predictive value for Aβ deposition.

**Conclusion:**

Integrating FW-DTI with structural and clinical features effectively differentiated Aβ positivity in AD, with FW-DTI indices contributing the highest predictive risks, demonstrating the potential of FW-DTI in AD diagnosis.

**Supplementary Information:**

The online version contains supplementary material available at 10.1186/s12880-026-02380-6.

## Introduction

Alzheimer’s disease (AD) is the most common cause of dementia, occurring most prevalently in the aged population [1]. Mild cognitive impairment (MCI) due to AD is a transitional stage between normal aging and AD. Early pathological hallmarks of AD, such as amyloid-beta (Aβ) deposition, may manifest during this stage [2]. In vivo detection of Aβ can be performed using cerebrospinal fluid measurement or positron emission tomography (PET) imaging. Aβ-PET is a non-invasive and more accessible alternative to cerebrospinal fluid measurement, but its use is limited by high costs and radiation exposure. Magnetic resonance imaging (MRI), widely used in AD research due to its non-invasive nature and broad accessibility, cannot directly detect amyloid-beta pathology. Therefore, further techniques are needed to identify Aβ-positive (Aβ+) and Aβ-negative (Aβ−) AD patients at an early stage.

Beyond amyloid deposition, cerebral small vessel disease–related vascular injury represents another important pathological mechanism in AD [3]. White matter lesions are the most common cerebral small vessel disease associated with AD [4]. Their alteration could precede neuronal loss during preclinical stages of the disease [5, 6], demonstrating its potential as a predictor of early histopathological changes in AD. Diffusion tensor imaging (DTI) is a widely used noninvasive modality in vivo to detect and longitudinally monitor changes in white matter integrity [7]. However, there is conflicting evidence regarding the distribution of impaired brain areas and characteristic structural alterations in DTI metrics [8]. Part of this variability may stem from the confounding influence of extracellular free water on tensor model fitting [9]. Free-water DTI (FW-DTI) can eliminate the partial volume effects caused by extracellular FW on conventional DTI in white matter by employing a two-compartment model to explicitly quantify changes in both tissue compartment and extracellular FW [10], thereby improving the sensitivity and accuracy of the analysis results. Compared with conventional DTI, FW-corrected DTI metrics may be more specific to white matter microstructural changes in AD [11], indicating that they may be more effective biomarkers of AD-related pathology, even in preclinical stages [12, 13].

Machine learning algorithms that can adequately model complex non-linear interactions among high-dimensional measures have been frequently employed in AD diagnosis [14–16]. Prior investigations have employed conventional DTI of white matter microstructural alterations with machine learning algorithms demonstrating the effectiveness of discriminating AD from MCI, and cognitively normal controls [17, 18]. However, the performance of MRI-based machine learning models for classifying AD and MCI exhibits considerable variation, with reported accuracy ranging from below 70% to over 99% [14, 19]. Importantly, the inherent technical limitations of DTI impede accurate characterization of complex water diffusion patterns, particularly because of sensitivity to the confounding effects of extracellular free water, resulting in suboptimal sensitivity of traditional DTI-based models for early disease screening.

Despite significant advances in the early diagnosis of AD in recent years, several important limitations remain. First, most prior studies did not incorporate Aβ status at enrollment [20], few have adopted Aβ positivity as an inclusion criterion, rendering models unable to distinguish AD from other types of dementia, particularly vascular dementia [21, 22]. In addition, current research often relies on cognitive impairment as the primary basis for disease staging and classification, while early pathological changes are frequently overlooked. This increases subjectivity and limits the diagnostic accuracy of these models [14]. The sensitivity of conventional DTI to extracellular free water contamination may limit its ability to precisely characterize subtle microstructural changes, highlighting the need for more advanced diffusion modeling approaches. MRI-based approaches combined with machine learning offer promising potential for predicting Aβ status [15, 16]. However, there is a lack of comprehensive evaluation of model performance in real-world clinical settings. Therefore, it is necessary to conduct further studies stratified by Aβ status and to integrate multimodal imaging data to enhance the accuracy, generalizability, and clinical utility of these models.

In the present study, we aimed to develop an FW-DTI-based machine learning model to differentiate AD with Aβ-PET identification. First, we compared performance across three machine learning methods—support vector machines (SVM), random forests (RF), and extreme gradient boosting (XGB)— to construct a diagnostic model for distinguishing Aβ deposition status in AD by integrating FW-DTI of white matter changes, gray matter imaging features, neuropsychological data, and other clinical information. Second, we incorporated SHapley Additive exPlanation (SHAP) value as an interpretable algorithm to identify influential characteristics based on the best-performing model, providing decision support for AD diagnosis and promoting the translation of neuroimaging research into clinical practice.

## Materials and methods

### Participants

We recruited 96 subjects from the Chinese PLA General Hospital between January 2019 and January 2024. Aβ PET negative cohort of MCI (MCI-n) and AD (AD-n) with 27 participants (MCI-n: *n* = 20, AD-n: *n* = 7) and Aβ PET positive cohort of MCI (MCI-p) and AD (AD-p) with 69 participants (MCI-p: *n* = 35, AD-p: *n* = 34) were included. To assess the generalization performance, 20% of the dataset was randomly selected to serve as an independent external test set.

MCI was diagnosed with the following criteria: subjective memory complaints, Clinical Dementia Rating (CDR) score of 0.5, a memory CDR ≥ 0.5, Mini-Mental State Examination (MMSE) score < 26, and no discernible impairment in activities of daily living [23]. Dementia due to AD was diagnosed with subjective memory complaints, MMSE < 26, CDR ≥ 1, and impaired activities of daily living [23]. Subjects with structural lesions were excluded [24].

Demographical, neuroimaging, and neuropsychological data and risk factors were collected. Clinical dementia assessment encompassed a thorough history, neurological examination, neuropsychological evaluation, brain MRI, molecular amyloid PET/CT scan, and laboratory tests. Laboratory tests included assessments of vitamin B_12_ levels, folate levels, renal function, hepatic function, thyroid function, thyroid-related antibodies, and screenings for sexually transmitted diseases. Neuropsychological evaluation included CDR, MMSE, Montreal Cognitive Assessment (MoCA), Auditory Verbal Learning Test (AVLT) memory test, and Stroop Color-Word Test executive test. Additionally, vascular risk factors, including diabetes, hypertension, hyperlipidemia, cerebrovascular disease, and psychiatric diseases, including anxiety and depression, were collected.

### Amyloid PET imaging

Aβ PET scans with ^18^F-Florbetaben or ^11^C-Pittsburgh compound-β (^11^C-PIB) tracers were acquired in all participants using a PET/CT (µMI 510 PET/CT, United Imaging, China) scanner in the Department of Nuclear Medicine, Chinese PLA General Hospital. Participants underwent a dynamic PET emission scan in 3D mode, configured with the following parameters: 120 kV, 110 mAs, and a slice thickness of 3 mm. Brain PET images were continuously collected over 20 min. A standard dynamic 40–70 min acquisition protocol following the intravenous injection of 370–555 MBq of ^11^C-PIB. ^18^F-Florbetaben was injected as an intravenous bolus of 300 ± 60 MBq, and images were obtained at 90–110 min post-injection.

For PIB PET, Aβ burden was evaluated by PIB standardized uptake value ratios (SUVR) within frontal, lateral temporal, anterior-posterior cingulate, parietal, and occipital cortex regions relative to uptake in the whole cerebellum for each subject [25], and PIB SUVR > 1.5 was identified as Aβ positivity [26]. For ^18^F-Florbetaben PET, Aβ status was assessed by three nuclear medicine experts (10-year, 6-year, and 4-years of Aβ PET reading experience, respectively) who were blinded to the clinical investigation results and diagnoses through visual interpretation according to the Brain Amyloid Plaque Load (BAPL) scores, with BAPL scores > 1 considered as positive Aβ deposition (1: no amyloid load, 2: minor amyloid load, and 3: significant amyloid load) [27].

### MR imaging acquisition

Multimodal MRI scans were acquired using a 3T scanner (Discovery MR750, GE Healthcare) with a 32-channel head coil at the Chinese PLA General Hospital. The data collection process utilized an array spatial sensitivity encoding technique, parallel imaging with a phase acceleration factor of *R* = 2 in the phase encoding direction. The MRI panel included T1-weighted and diffusion-weighted MRI scans. T1 scans were acquired using an axial 3D fast spoiled gradient echo sequence with the following parameters: repetition time (TR) = 6.6 ms, echo time (TE) = 2.9 ms, inversion time = 450 ms, flip angle = 12°, acquisition matrix = 256 × 256, reconstruction matrix = 512 × 512, field of view (FOV) = 256 × 256 mm^2^, voxel size = 0.5 × 0.5 × 1 mm^3^, and slices = 172. The diffusion acquisition protocol included an echo planar imaging sequence with the following parameters: direction = 30, TR/TE = 11,500/104.1 ms, flip angle = 90°, acquisition matrix = 224 × 224, reconstruction matrix = 512 × 512, FOV = 240 × 240 mm^2^, voxel size = 0.94 × 0.94 × 2 mm^3^, slices = 70, *b*-value = 1000s/mm^2^, *b*_0_ images = 1.

### MR image preprocessing

#### T1 image processing

T1 image processing was performed using the CAT12 (https://neuro-jena.github.io/cat//) [28] toolbox implemented in SPM12 (https://www.fil.ion.ucl.ac.uk/spm/) [29] and included skull stripping, intensity normalization, subcortical segmentation, cortical parcellation, and region-of-interest (ROI) labeling. Voxel-based morphometry (VBM) and surface-based morphometry (SBM) were used to examine the structural MRI data. T1 data first underwent brain tissue segmentation using an adaptive maximum posterior algorithm to segment the brain into gray matter, white matter, and cerebrospinal fluid tissue maps. The volumetric measure for each tissue on the Neuromorphometrics atlas was acquired after the data were aligned to the MNI152 standard space. After completing the voxel-based processing, the cortical thickness estimation and center surface reconstruction were conducted using a projection-based thickness method. Cortical thickness, depth, gyrification, and fractal dimension indices of the SBM analysis were calculated in 152 total ROIs using the aparc_a2009s atlas. Reconstructed output files were visually inspected to rule out subcortical segmentation errors before analysis.

#### Diffusion FW image processing

Diffusion MRI data were preprocessed to remove non-brain tissue, correct head motion, eddy-current-induced distortion, and intensity bias using functional MRI of the brain (FMRIB) software library (FSL) [30] and a pipeline for analyzing brain diffusion images (PANDA, www.nitrc.org/projects/panda) [31]. We extracted a brain mask from the unweighted *b*_0_ volume for each participant. Subsequently, standard DTI index images were generated by fitting the single-tensor diffusion model. Utilizing the preprocessed diffusion data, we calculated diffusion metrics, including fractional anisotropy (FA), mean diffusivity (MD), axial diffusivity (AxD), and radial diffusivity (RD).

A single-shell FW estimation model was performed using the DIPY package in Python [32]. Based on the assumption that the diffusion signal within each voxel originates from the coexistence of the free water region and the tissue region, the signal of each voxel was modeled using a two-compartment method, resulting in both FW (isotropic tensor with fixed diffusion constant of water at 37 °C) map and the FW-corrected tissue tensor map [33]. Subsequently, individual tissue-specific FW-DTI indices, including tissue fractional anisotropy [FAt], tissue mean diffusivity [MDt], tissue radial diffusivity [RDt], and tissue axial diffusivity [AxDt], were calculated by removing the FW compartment. Finally, FW-DTI and conventional DTI indices were calculated in specific ROIs, respectively, including 48 regions from the ICBM-DTI-81 white-matter labels atlas.

### Machine learning modeling approaches

With Aβ-PET as the gold standard, multimodal image data, and clinical characteristics as input variables, three supervised machine learning algorithms were applied to construct the MCI and AD binary diagnosis models of Aβ + and Aβ-. Three supervised machine learning algorithms, including RF, SVM, and XGB, were used utilizing scikit-learn [34] and xgboost [35] packages in Python. A feature selection algorithm was incorporated into the RF and SVM algorithms to select the top 5 crucial features for preliminary screening, and the process was encapsulated in a pipeline to prevent data leakage.

An independent external dataset including 18 patients (13 Aβ + and 5 Aβ-) was retrieved, while an internal dataset comprising 78 subjects was selected and divided into a training set splitting into the training and validation set, and a test set with a ratio of 8:2. Classification effects were evaluated with single and multimodal machine learning models with inputs as follows: single FW-DTI; FW-DTI and clinical features; FW-DTI, clinical features, and VBM; FW-DTI, clinical features, and SBM. In the model, clinical features included cognitive function and demographic characteristics. We utilized a GridSearch approach to select the optimal hyperparameters and the accuracy rate as an assessment measure. To ensure reproducibility, model hyperparameters were optimized using GridSearchCV. For the RF model, n_estimators was searched within [150, 200, 600], max_features within [0.1, 0.5, 1], and min_samples_split within [0.005, 0.1, 0.5]. For the SVM, a linear kernel was used with C = 5 and gamma = 0.1, determined based on preliminary experiments. For the XGBoost model, max_depth was searched within [1, 3, 5, 10], learning_rate within [0.1, 0.25, 0.5, 1], and n_estimators within [100, 150, 200]. Detailed parameter selection information can be found elsewhere in the Supplemental material.

**Fig. 1:**
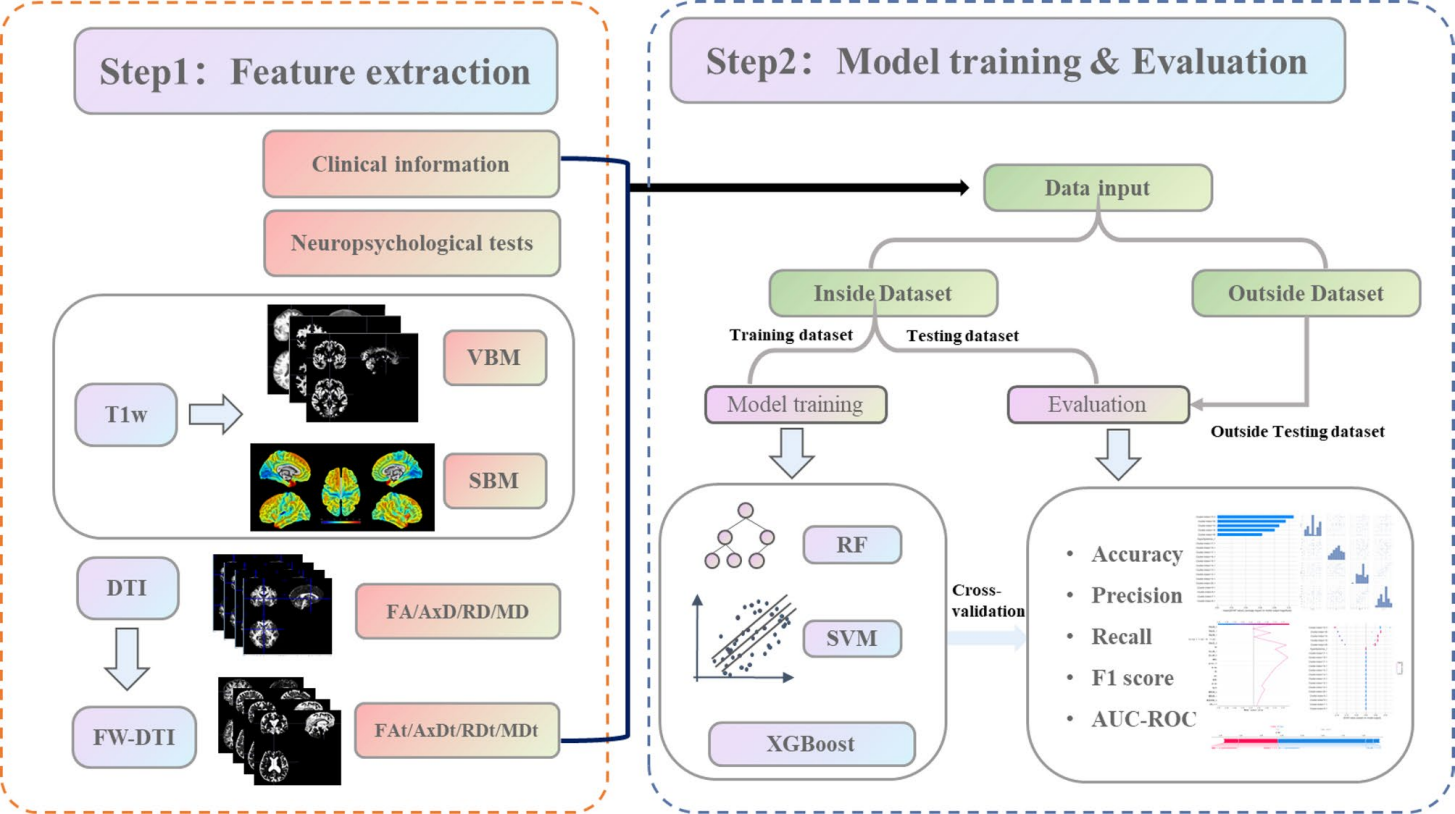
The flow chart of the study. RF = random forest; SVM = support vector machines; XGB = extreme gradient boosting; voxel-based morphometry = VBM; surface-based morphometry = SBM

We removed measures with a proportion of missing values ≥ 20% in each cohort or each measure and imputed missing values using the mode of measures. Z-score was adopted for continuous measurement, and one-hot for categorical measures, respectively. The training data were processed using the BorderlineSMOTE [36] algorithm to correct the issue. This technique enhances the data distribution by generating new samples and amplifying the number of samples from a limited number of bordering class samples. The flowchart of the research is revealed in Fig. 1.

### Interpretability and assessment of validation

We employed a 5-fold cross-validation method in the internal cohort. The performance of the model was evaluated, including accuracy, precision, recall, and F1 score. The efficiency was also assessed using the area under the receiver operating characteristic curve (AUC-ROC) and confusion matrix. The machine learning algorithm with the highest model performance was chosen as an optimal model and subsequently tested on both an internal test cohort and an external test set. To enhance the interpretability of machine learning models, SHAP was used to interpret model predictions [37]. Influential variables were ranked by calculating the absolute mean SHAP value using the shap package [37] in Python. Each feature’s marginal contribution was measured, and a bar chart of feature contribution was plotted. The top 5 features were visualized for RF and SVM models, while the top 20 features were extracted for XGB models.

### Statistical analysis

All the statistical analysis was performed using SPSS Statistics, Version 25.0 (IBM). Continuous variables are presented as Mean ± SD, and categorical variables are presented as counts for each category. To verify the group differences based on radiomics features, a Chi-squared test was performed on sex, psychiatric disorders, and vascular risk factors. At the same time, a one-way analysis of variance was conducted specifically for sex and education. A one-way analysis of covariance (ANCOVA) was employed to test group differences in neuropsychological performance (with age, sex, and education as covariates), followed by *post hoc* testing and Bonferroni correction for multiple comparisons at *p* < 0.05.

## Results

### Demographic and clinical characteristics

We recruited 96 elderly participants. Table 1 presented demographic and clinical information for 20 MCI-n, 7 AD-n, 35 MCI-p, and 34 AD-p participants. There were no significant differences in age (*p* = 0.247), gender (*p* = 0.830), education (*p* = 0.336), vascular risk factor (*p* = 0.529), and psychiatric disorders (*p* = 0.256) among groups. All neuropsychological tests showed significant differences among groups. AD-p and AD-n patients had worse cognitive performance than MCI-p and MCI-n in general cognitive abilities (CDR, MMSE, and MoCA), memory (AVLT N1-N3, N4, N5, N1-N5), and executive function (AD-p > MCI-p/MCI-n). Patients with MCI showed significantly higher AVLT scores compared with AD patients in the Aβ-positive subgroup, including N1–N3 (*p* < 0.001), N4 (*p* = 0.001), N5 (*p* = 0.002), and N1–N5 (*p* < 0.001). In the Aβ-negative subgroup, significantly higher scores were observed in MCI-n compared with AD-n for N5 (*p* = 0.028) and N1–N5 (*p* = 0.038), whereas differences in N1–N3 and N4 did not reach statistical significance. Additionally, the same results were identified in N1-N5 (*p* = 0.038) in Aβ negative patients (MCI-n > AD-n).

**Table 1:** Demographical and neuropsychological characteristics of participants

	MCI-*n* (*n* = 20)	MCI-*p* (*n* = 35)	AD-*n* (*n* = 7)	AD-*p* (*n* = 34)	F/c^2^	*p*	Post Hocs ^a^
***Demographical characteristics***							
Sex(F/M)^b^	10/10	14/21	3/4	17/17	0.88	0.830	-
Age ^c^	71.00 ± 8.16	75.24 ± 8.19	71.86 ± 2.54	72.42 ± 8.59	1.40	0.247	-
Education ^c^	11.93 ± 3.30	12.86 ± 3.69	13.00 ± 3.51	11.43 ± 3.32	1.14	0.336	-
Psychiatric disorders(1/0)^b^	4/16	4/31	3/4	6/28	2.31	0.256	-
Vascular risk factor(1/0)^b^	15/5	28/7	7/0	26/8	2.22	0.529	-
***Neuropsychological characteristics***							
CDR ^d^	0.50 ± 0.16	0.50 ± 0.00	1.14 ± 0.38	1.24 ± 0.48	41.72	<0.001	MCI-n < AD-n / AD-p; MCI-p < AD-n / AD-p
MMSE ^d^	24.85 ± 2.92	24.71 ± 2.54	21.29 ± 2.50	19.24 ± 4.36	18.60	<0.001	MCI-n > AD-n / AD-p; MCI-p > AD-p
MoCA ^d^	21.25 ± 3.87	21.14 ± 3.08	15.14 ± 4.56	14.29 ± 4.62	22.36	<0.001	MCI-n > AD-n / AD-p; MCI-p > AD-n / AD-p
Stroop C-B time ^d^	84.62 ± 50.95	136.63 ± 110.63	97.57 ± 48.88	122.03 ± 93.16	1.70	0.216	-
AVLT ^d^							
N1-N3	12.55 ± 4.43	12.51 ± 3.84	9.00 ± 3.00	8.82 ± 2.26	8.08	<0.001	MCI-n / MCI-p > AD-p
N4	3.20 ± 2.17	1.97 ± 2.37	1.43 ± 1.27	0.29 ± 0.68	11.49	<0.001	MCI-n > AD-p; MCI-p > AD-n /AD-p
N5	2.45 ± 2.09	1.54 ± 2.12	0.43 ± 0.79	0.09 ± 0.29	10.31	<0.001	MCI-n > AD-n / AD-p; MCI-p > AD-p
N1-N5	18.20 ± 8.26	15.54 ± 8.19	10.43 ± 4.47	9.21 ± 2.32	9.78	<0.001	MCI-n > AD-n / AD-p; MCI-p > AD-p

Participants’ information is presented as mean ± standard deviation

MCI-n = mild cognitive impairment with negative amyloid-beta (Aβ); MCI-p = mild cognitive impairment with positive Aβ; AD-n = Alzheimer’s disease with negative Aβ; AD-p = Alzheimer’s disease with positive Aβ; F = female; M = male; CDR = Clinical Dementia Rating scale; MMSE = Mini-Mental State Examination; MoCA = Montreal Cognitive Assessment; AVLT = Auditory Verbal Learning Test

a. Post hoc tests based on estimated marginal means adjusted for covariances and Bonferroni correction for multiple comparisons. Only significant contrasts are presented. Significance was set at *p* < 0.05

b. Chi-squared (χ2) test

c. ANOVA test, post hoc tests, and Bonferroni correction for multiple comparisons

d. ANCOVA test (age-, sex-, and education-corrected)

### Classification performance comparison between FW-DTI single-modality and multi-modality among three different machine learning approaches for distinguishing AD and MCI with positive Aβ from negative Aβ

Classification performance and comparisons based on FW-DTI single-modality and multi-modality among three different machine learning models for distinguishing Aβ statuses of MCI and AD were presented in Table 2. SVM model demonstrated strong performance when utilizing only the FW-DTI features for unimodal analysis, achieving an accuracy of 0.867 and an AUC-ROC score of 0.864 on the internal test set. Additionally, the SVM model (accuracy, 77.8%; F1 score, 86.7%; AUC-ROC, 0.785) outperformed RF (accuracy, 77.8%; F1 score, 85.7%; AUC-ROC, 0.692) and XGB (accuracy, 72.2%; F1 score, 82.8%; AUC-ROC, 0.554) models on the external test. Incorporating clinical features, including neuropsychological and demographic characteristics, and structural MRI features, including VBM or SBM, into the machine learning model resulted in higher classification performance. Incorporating VBM, FW-DTI and clinical features as inputs, the XGB model outperformed the RF and SVM, achieving an accuracy of 77.8%, an F1 score of 86.7%, and AUC-ROC values of 0.831 on the external test set. When cortical thickness, depth, gyrification, and fractal dimension of the SBM cortical indices, combined with FW-DTI and clinical features used as inputs, the AUC in the internal test set was higher than 0.816, and the AUC value of XGB was up to 0.977 compared with the other two models. However, in the external test set, AUC values were relatively low across all three models, indicating limited generalization performance compared with other multimodal classification models.

**Table 2:** Classification performance comparison between FW-DTI single-modality and multi-modality among three different machine learning approaches for distinguishing AD and MCI with positive Aβ from negative Aβ

Features	Classifier	Inside testing cohort					Outside testing cohort				
		Accuracy	Precision	Recall	F1 score	AUC-ROC score	Accuracy	Precision	Recall	F1 score	AUC-ROC score
FW-DTI	RF	0.800	0.900	0.818	0.857	0.727	0.778	0.800	0.923	0.857	0.692
	SVM	0.867	1.00	0.818	0.900	0.864	0.778	0.765	1.00	0.867	0.785
	XGB	0.800	0.833	0.909	0.870	0.864	0.722	0.750	0.923	0.828	0.554
FW-DTI + clinical features	RF	0.847	0.886	0.909	0.897	0.730	0.778	0.765	1.00	0.867	0.724
	SVM	0.867	1.00	0.818	0.900	0.932	0.722	0.722	1.00	0.839	0.708
	XGB	0.867	0.846	1.00	0.917	0.955	0.722	0.722	1.00	0.839	0.769
FW-DTI + clinical features + VBM	RF	0.867	1.00	0.818	0.900	1.000	0.722	0.750	0.923	0.828	0.692
	SVM	0.933	0.917	1.00	0.957	0.955	0.778	0.765	1.00	0.867	0.738
	XGB	0.867	0.846	1.00	0.917	0.932	0.778	0.765	1.00	0.867	0.831
FW-DTI + clinical features + SBM	RF	0.800	0.900	0.818	0.857	0.816	0.778	0.765	1.00	0.867	0.408
	SVM	0.893	1.00	0.855	0.920	0.930	0.722	0.722	0.985	0.839	0.662
	XGB	0.867	0.909	0.909	0.909	0.977	0.833	0.813	1.00	0.897	0.631

Clinical features include neuropsychological and demographic features. RF = random forest; SVM = support vector machines; XGB = extreme gradient boosting; VBM = voxel-based morphometry; SBM = surface-based morphometry

To further evaluate classification performance under class imbalance, sensitivity and specificity were calculated for these models. The RF and XGB models achieved high sensitivity (both 1.0) but limited specificity (ranging from 0.2 to 0.4), indicating a reduced ability to correctly identify Aβ-negative subjects. Notably, SBM-based models demonstrated a marked performance decline in the external test set, indicating potential overfitting and sensitivity to dataset-specific patterns. This issue is likely exacerbated by the limited sample size and high feature dimensionality. Consequently, the results derived from SBM features should be interpreted with caution, as their generalizability appears inferior to that of VBM- and FW-DTI-based models.

### Classification performance of standard DTI and clinical metrics

To compare performance, we evaluated multiple machine learning models using conventional DTI metrics and clinical features **(**Table 3). Compared with FW-DTI single-modality, the results of conventional DTI characteristics as input showed worse classification performance and stability. FW-DTI obtained accuracies all above 80% among three different machine learning approaches on the internal dataset (RF = 0.800, SVM = 0.867, XGB = 0.800), while conventional DTI had accuracy all below 0.787 (RF = 0.720, SVM = 0.787, XGB = 0.733). The F1 scores for the standard DTI models were also lower than those for the FW-DTI models.

**Table 3:** Classification performance comparison between conventional/ DTI and clinical features among three different machine learning approaches for distinguishing AD and MCI with positive Aβ from negative Aβ

Feature	Classifier	Inside testing cohort						Outside testing cohort					
		Accuracy	Precision	Recall	F1 score	AUC-ROC score		Accuracy		Precision	Recall	F1 score	AUC-ROC score
Clinical features	RF	0.840	0.945	0.836	0.880	0.905		0.633		0.764	0.715	0.737	0.501
	SVM	0.747	0.919	0.727	0.808	0.876		0.372		0.640	0.277	0.384	0.511
	XGB	0.733	0.889	0.727	0.800	0.886		0.556		0.692	0.692	0.692	0.738
DTI	RF	0.720	0.809	0.809	0.809	0.709		0.778		0.765	1.00	0.867	0.729
	SVM	0.787	0.950	0.745	0.835	0.914		0.667		0.789	0.738	0.643	0.637
	XGB	0.733	0.818	0.818	0.818	0.614		0.722		0.786	0.846	0.815	0.815

Clinical features include neuropsychological and demographic features. RF = random forest; SVM = support vector machines; XGB = extreme gradient boosting

The clinical features of single-modality yielded poor overall performance on the external test set compared to standard DTI and FW-DTI. The accuracy of external test sets of SVM, RF, and XGB was only 37.2%, 55.6%, and 63.3%, respectively. Also, the generalization and stability were poor, with an AUC-ROC value between 0.501 and 0.738.

### Interpretability of the FW-DTI multi-modality machine learning model and feature importance

**Fig. 2:**
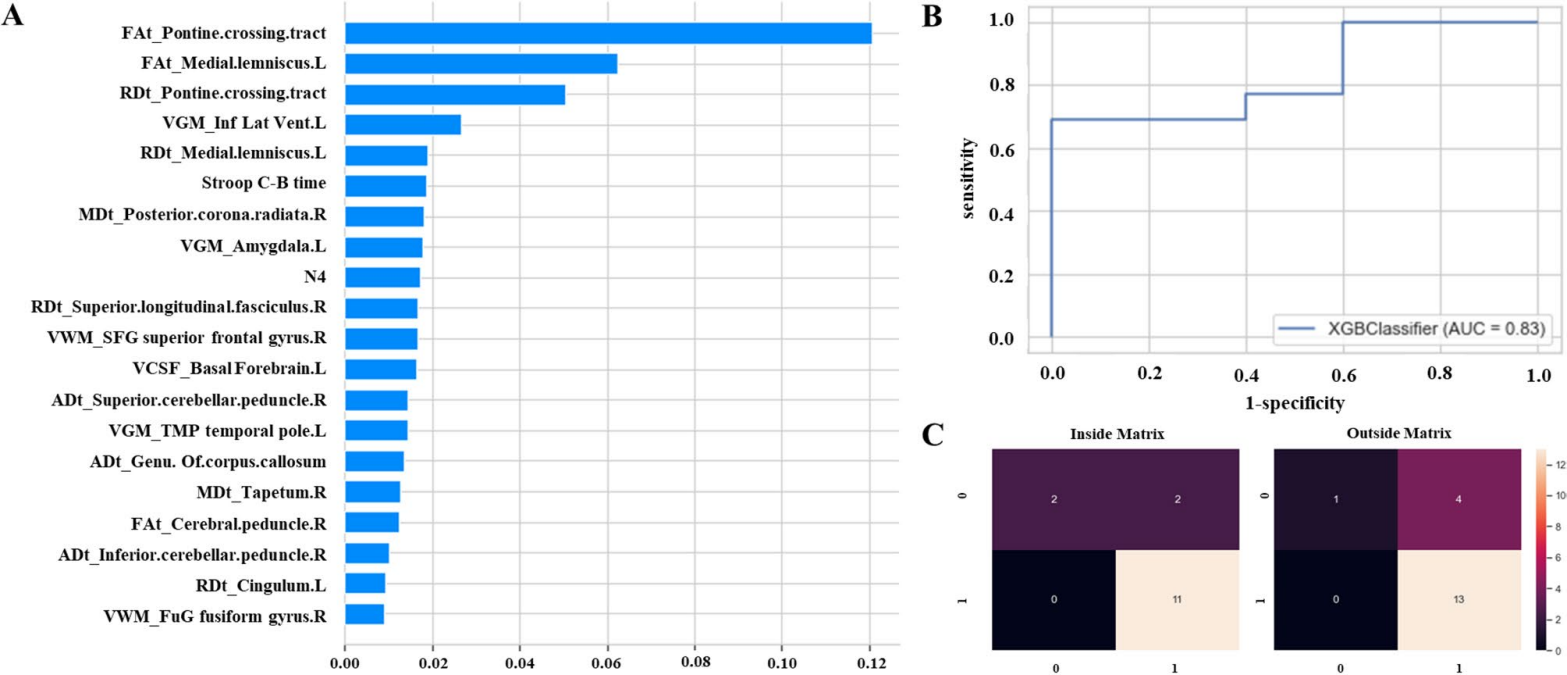
Performance evaluation and visualization results for the best XGB model using multimodal data with FW-DTI, VBM and clinical information as inputs. (**A**) Top 20 influential input features from XGB. The histogram showed each feature’s contribution to the model, with the horizontal axis representing each feature’s mean absolute SHAP value. (**B**) Receiver operating characteristic curve curves and area under the curves (AUC) values for external test sets, with 1-specificity on the horizontal axis and sensitivity on the vertical axis. (**C**) The left panel displays the confusion matrix for the internal test set, while the right panel shows the confusion matrix for the external test set

Confirming that FW-DTI, VBM, and combining clinical features with XGB achieved the best performance compared to other models, we adopted SHAP value, ROC line, and confusion matrix to explore the contributions of indices and their associated brain regions to the model performance. Figure 2A showed the 20 most influential variables identified by the XGB model, with FW-DTI indices emerging as the most critical feature in the optimal model. As expected, FW-DTI indices were shown to have the highest predictive risk of Aβ deposition, including FAt and RDt values of the pontine crossing tract and the FAt values of the left medial lemniscus. Additionally, Stroop C-B time and AVLT scores were selected as the critical elements of cognitive decline for discriminating Aβ statuses in AD. Structural imaging indices, such as grey matter volumes in the left inferior lateral ventricle and the left amygdala region, could also be interpreted as risk factors for Aβ deposition.

Meanwhile, the XGB model achieved an outstanding performance with an AUC value of 0.83 for the external test set (Fig. 2B). Analysis of the confusion matrices for internal and external test sets revealed that the model demonstrated higher sensitivity for Aβ + cases, correctly identifying 11 and 13 true positives, respectively. In contrast, fewer Aβ- cases were classified accurately (Fig. 2C).

## Discussion

In this study, we developed interpretable machine learning classification models of Aβ status in AD with FW-DTI indices, structural VBM and SBM features, and clinical features. Moreover, we investigated the contribution of classification characteristics by calculating SHAP values. The key findings of the present study were as follows. First, FW-DTI indices demonstrated great classification performance of Aβ positivity in both the internal and external test sets in the single-modality machine learning model. Second, FW-DTI, which incorporates demographic and neuropsychological clinical features, and VBM structural MRI within a multimodal XGB framework, further enhanced diagnostic accuracy. Third, FW-corrected DTI indices, including FAt and RDt values with higher SHAP values, were most closely associated with Aβ deposition. These findings suggested that the combination FW-DTI technique with machine learning algorithms may provide a valuable strategy for identifying Aβ pathology in AD.

The most obvious finding to emerge from the data-driven analysis is that FW-DTI indices are an effective imaging biomarker for Aβ deposition status classification. This finding aligns with the established common finding of white matter microstructural decline in AD [4]. White matter comprises the axons of cortical neurons, plays a critical role in the propagation of nerve impulses, and in maintaining brain-wide communication. Critically, FW-DTI imaging has been proposed to explicitly quantify changes in both tissue compartment and extracellular FW, thereby effectively removing the impact of the partial volume effect associated with extracellular FW in white matter [10]. Furthermore, the fractional volume of FW, which is thought to represent isotropic water transport in the interstitial extraneuronal space, may be more sensitive to mild vascular problems in apparently normal tissue, including neuroinflammation [38, 39], axonal injury, demyelination [40], and blood-brain barrier permeability modulation [41]. Previous studies have explored the association between FW-DTI metrics and Aβ accumulation, suggesting that FW-DTI sensitively reflects changes in white matter microstructure closely linked to abnormal Aβ deposition [42–45].

As expected, FW-DTI single-modality performs a more sensitive classification than conventional DTI in the dependent external test set across various machine learning models. This finding is consistent with previous mechanistic studies demonstrating that FW-DTI, with reduced bias from partial volume effect, can more objectively reflect changes in white matter microstructure [46, 47]. Moreover, our study confirms the high sensitivity of FW-DTI for identifying Aβ deposition compared to conventional DTI indices from a data-driven perspective, reinforcing the reliability of FW-DTI indices for Aβ status classification. Our models classified Aβ positivity utilizing FW-DTI indices in an internal test dataset, achieving an accuracy of approximately 75%, consistent with previously reported studies [48], thereby further substantiating the biological specificity advantage of FW-DTI metrics in pathological staging, similar to the accuracy reported in previous machine learning studies [49]. 

Despite considerable research advances, the impact of these studies on clinical practice remains limited, due to reliance on single-modality analyses; this narrow focus may ignore valuable information from other imaging modalities. It has also been demonstrated that using multi-model neuroimaging data types results in greater accuracy than using only one [7]. In our study, multi-model features enhanced classifier performance compared to FW-DTI single modality. This finding aligned with a previous study that incorporated standard DTI [50]. AD progression is a complex process best captured by integrating multiple considerations, while complementary information of different modalities can be combined [51, 52]. Some studies have suggested that neuropsychological scales are helpful for the classification and diagnosis of AD [53, 54]. However, collecting and measuring neuropsychological tests and performing PET imaging is highly challenging, and they are resource-intensive, time-consuming, and expensive. It is necessary to consider the economic and time costs of examination to minimize the medical burden on patients. The classification features of FW-DTI and VBM are both based on MRI, and the cognitive scales AVLT and Stroop are relatively simple and economical, which is more likely to serve clinical purposes in the future.

It is important to note that this research aimed to achieve improved Aβ status classification and gain insights into the influential factors driving classifier decision-making. SHAP value can provide global explanations for machine learning models, helping clarify the factors influencing decision-making. This interpretability overcomes the often-criticized lack of transparency in classification models, allowing for the identification of critical components by mapping the importance of imaging and histological features. Our results highlighted that FW-DTI indices in the posterior thalamic radiation, pontine crossing tract, and medial lemniscus were the most affected features in classification models utilizing FW-DTI and cognitive psychological scales. Recent studies have suggested that Aβ pathology follows a stage-dependent progression, with early alterations involving distributed cortical and subcortical networks, accompanied by microstructural white matter changes [55]. In AD, the posterior thalamic radiation and medial lemniscus, as critical projection fiber tracts, may undergo pathological changes driven by Aβ deposition even in the preclinical stage of the disease [4, 56].

Previous mechanistic investigations have demonstrated that thalamic connectivity is disrupted in AD, with early-stage alterations particularly evident in the posterior thalamic radiation [57]. These changes manifest as increased fiber‐orientation dispersion accompanied by a biphasic pattern of decreased FA and increased MD [58, 59]. Microstructural injury of posterior thalamic radiation fiber tracts may underlie deficits in visuospatial integration and contextual memory observed in patients [60]. In this context, the involvement of pontine crossing tract and medial lemniscus may reflect early or transitional white matter vulnerability associated with Aβ-related disease progression, although the exact temporal sequence requires further investigation [61]. Notably, this pattern of white matter degeneration aligns closely with the data‐driven features identified in our FW-DTI classification model, further corroborating its robustness and suggesting that impaired thalamo‐cortical information transfer efficiency may serve as a critical early marker of AD pathological progression. Therefore, FW-DTI tissue indices within these projection fiber tracts could indicate an early neurodegenerative decline.

Consistent with multimodal diagnostic frameworks, combining features reflecting brain atrophy, FW-DTI white matter microstructural alterations, and neuropsychological measurements improves the accuracy of Aβ status classification. In the FW-DTI combined clinical features and the FW-DTI combined VBM and clinical features models, overlapping influential factors emerged alongside FW-DTI indices. These indices included gray matter volume in the inferior lateral ventricle, which reflects cerebral atrophy, and N4 and Stroop C-B times. Changes in gray matter volume in this region may be linked to AD progression, as brain tissue atrophy leads to a relative enlargement of the ventricular system. Consistent with previous studies, AVLT and Stroop metrics reflect memory and attention functions in AD patients, which are crucial aspects associated with AD pathology [62, 63].

We found substantial potential of RF, SVM, and XGBoost models for accurate Aβ diagnosis, which is consistent with previously reports [64]. As an integrated learning method, RF constructs plenty of decision trees while training the data and fixes the overfitting issue when using a single decision tree [65]. SVM works by determining the optimal hyperplane to optimize the maximizes between different classes, performing well even for high-dimensional data [66]. Notably, oversampling algorithms exhibited superior stability and robustness compared to the SVM classifier when handling multimodal datasets, while SVM outperformed other approaches in smaller sample datasets [14, 67]. Partly similarly to previous study, RF and XGB achieved better classification in risk assessment and effectively managed unbalanced data [68], rendering them well-suited for the complexities of medical diagnosis and handling imbalanced situations. For unbalanced data, the XGB algorithm achieves performance by integrating multiple weak classifiers and adjusting sample distribution in iterations [69]. Besides, SHAP is a model-independent interpretation technique that helps explain the results of a predictive model. The interpretation is based on the SHAP value for each feature, representing a feature’s contribution to predicting the risk of complications. The final prediction result can be interpreted through the SHAP value for all features and the average prediction result. The application of these complementary algorithms strengthens the stability and generalizability of the predictive models.

The present study showed that multimodal models incorporating SBM cortical, FW-DTI metrics, and clinical features achieved high performance in the internal test set, particularly for the XGB model. However, the marked decline in performance in the external test set suggests limited generalizability and raises the possibility of model overfitting. Several factors may contribute to this phenomenon. First, the relatively modest sample size, especially within subgroup analyses, may increase the risk of the model capturing dataset-specific patterns rather than disease-related features. Second, the high dimensionality of multimodal imaging features may further amplify model complexity despite the use of machine learning regularization strategies. Third, potential distribution differences between the internal and external datasets may also affect model transferability. To mitigate overfitting, we implemented multiple strategies, including cross-validation during model training, independent test set evaluation, and algorithm-level regularization within RF, SVM, and XGB frameworks. Nevertheless, these measures may not fully eliminate overfitting in small-sample, high-dimensional settings. Future studies with larger, multi-center cohorts and external prospective validation are warranted to improve robustness and generalizability.

Most research on machine learning models focused on the technical specifics, often overlooking clinical integration and practical application [70]. The status of Aβ accumulation was a strength of the study, while previous AD classification studies concentrated on the subtype identification. Additionally, due to the limitations of the techniques, neuropsychological assessments remain the primary protocol for AD, particularly in its early stages. However, cognitive tests vary significantly between individuals because of investigator bias [71]. Additionally, a lack of validation for Aβ has resulted in approximately 40% misdiagnosis in classification studies [72]. In previous studies, Aβ accumulation in individuals with MCI or AD has not been confirmed strictly [73, 74], raising the possibility that cognitive decline could be attributed to other etiologies. To address this issue, amyloid status was determined using established PET tracers. Both ¹¹C-PiB and ¹⁸F-florbetaben have demonstrated high concordance in binary Aβ classification, supporting the reliability of the biomarker definition in the present cohort [75].

This study has some limitations. First, the relatively modest sample size may limit the robustness of the findings; larger, well-characterized clinical cohorts will be established in future work to further validate the proposed models. Second, the lack of Aβ- data in our dataset created an imbalance in the training data, leading classification models to exhibit a more substantial bias toward recall over precision, thus enhancing the differentiation of positive cases [76]. Although we applied the BorderlineSMOTE algorithm to address this issue, further inclusion of negative data is necessary for a comprehensive analysis. Although the models achieved high sensitivity, their low specificity indicates a limited ability to correctly identify Aβ-negative cases. This suggests that the models are biased toward positive predictions and may therefore be more appropriate for screening purposes than for definitive classification. Third, two amyloid PET tracers were used in this study. Although both tracers have demonstrated high concordance in determining binary Aβ status, potential inter-tracer variability cannot be completely excluded. This issue should be further examined in future studies with complete tracer metadata and larger cohorts. Finally, the study was cross-sectional, future longitudinal datasets will be needed for in-depth analysis and discussion.

## Conclusion

This study established a machine learning framework integrating FW-DTI with VBM and clinical features to differentiate Aβ status. Further influential analysis indicated that FW-DTI features can effectively predict amyloid status, suggesting their potential as neuroimaging biomarkers for detecting preclinical AD.

## Electronic supplementary material

Below is the link to the electronic supplementary material.

Supplementary Material 1

## Data Availability

The datasets generated and analyzed during the current study are not publicly available due to patient privacy and institutional ethical restrictions, but are available from the corresponding author on reasonable request, subject to approval by the relevant ethics committee.

## Abbreviations

^11^C-PIB: ^11^C-labeled Pittsburgh Compound-B
^18^F-Florbetaben: ^18^F-labelled florbetaben
AD: Alzheimer’s disease
AUC-ROC: Area Under the Receiver Operating Characteristic Curve
AVLT: Auditory Verbal Learning Test
Aβ: Amyloid-beta
CDR: Clinical Dementia Rating
DTI: Diffusion tensor imaging
FA: Fractional anisotropy
FN: False Negatives
FP: False Positives
FPR: False Positive Rate
FSL: FMRIB Software Library
MCI: Mild cognitive impairment
MMSE: Mini-Mental State Examination
MoCA: Montreal Cognitive Assessment
MRI: Magnetic Resonance Image
PET: Positron emission tomography
RF: Random forest
SUVR: Standardized uptake value ratios
SVM: Support vector machines
TN: Ture Negatives
TP: True Positives
TPR: True Positive Rate
VBM: Voxel-based morphometry
XGBoost: Extreme gradient boosting
SHAP: Shapely Additive exPlanations
