# Supplementary material for: Amyloid-beta statuses prediction with free water MR imaging features in Alzheimer’s disease using machine learning models
Source: BMC Med Imaging. 2026 Apr 27;26:222. doi: 10.1186/s12880-026-02380-6 (PMC13130614; doi:10.1186/s12880-026-02380-6)
Supplement: Supplementary file 1 — Supplementary Material 1 [file 12880_2026_2380_MOESM1_ESM.docx]

**Supplementary Material**

Hyperparameter optimization was performed separately for each algorithm using grid search within a cross-validation framework on the training set. Accuracy was used as the primary scoring metric during the search process. Stratified K-fold cross-validation was adopted to preserve the class distribution in each fold. All random processes were seeded to ensure reproducibility.

1. Random Forest (RF)

We used a RandomForestClassifier (random_state=38, n_jobs=-1). The following hyperparameters were tuned using grid search with 10-fold stratified cross-validation (random_state=123, shuffle=True): n_estimators: [200, 150, 600], max_features: [1, 0.1, 0.5], min_samples_split: [0.005, 0.1, 0.5].

2. Support Vector Machine (SVM)

We used an SVC (probability=True, random_state=123) combined with SelectKBest(mutual_info_classif, k=5) for feature selection. Hyperparameters were tuned using grid search with 5-fold cross-validation: C: [0.01, 0.1, 0.5, 1, 1.5, 2, 5, 10], gamma: [0.1, 1, 10] (included in the grid but not applicable for linear kernel).

3. Extreme Gradient Boosting (XGBoost)

We used an XGBClassifier(). Hyperparameters were tuned using grid search with 10-fold stratified cross-validation (random_state=38, shuffle=True): max_depth: [1, 3, 5, 10], learning_rate: [0.1, 1, 0.25, 0.5], n_estimators: [100, 150, 200].
